# Recombinant FIX Fc fusion protein activity assessment with the one‐stage clotting assay: A multicenter, assessor‐blinded, prospective study in Japan (J‐Field Study)

**DOI:** 10.1111/ijlh.13133

**Published:** 2019-12-10

**Authors:** Katsuyuki Fukutake, Tomomi Kobayashi, Jurg M. Sommer, Toshiyuki Hirakata

**Affiliations:** ^1^ Department of Laboratory Medicine Tokyo Medical University Tokyo Japan; ^2^ Department of Molecular Genetics of Coagulation Disorders Tokyo Medical University Tokyo Japan; ^3^ Sanofi K. K. Rare Blood Disorders Medical Sanofi Genzyme Medical Tokyo Japan; ^4^ Sanofi Waltham MA USA; ^5^ Biogen Japan Inc. Tokyo Japan

**Keywords:** biological assay, factor IX, hemophilia B, Japan, recombinant FIX Fc fusion protein

## Abstract

**Introduction:**

The one‐stage clotting assay is used to measure factor IX (FIX) activity in patients’ plasma samples and in FIX products for hemophilia treatment. However, the diversity of reagents and instruments has resulted in significant FIX assay variability.

**Methods:**

The accuracy of the one‐stage clotting assay to measure recombinant FIX Fc fusion protein (rFIXFc) activity was evaluated by major Japanese hemophilia treatment centers and commercial laboratories that measure factor IX activity for a majority of hemophilia B patients in Japan. Plasma‐derived FIX (pdFIX) and recombinant FIX (rFIX) products were used as comparators. FIX‐deficient plasma was spiked with four levels of FIX products based on label potency and measured under blinded conditions by routine one‐stage clotting assay procedures in 19 participating laboratories. Interlaboratory coefficient of variation and spike recovery were calculated.

**Results:**

Interlaboratory coefficient of variation of rFIXFc was not significantly different from that of rFIX, but appeared larger than that of pdFIX. Mean spike recovery for rFIXFc was generally comparable to rFIX and pdFIX. However, larger discrepancies between pdFIX and rFIX were observed in three of nine laboratories using ellagic acid‐based activated partial thromboplastin time reagents.

**Conclusion:**

Recombinant FIX Fc fusion protein activity was found to be similar to that of rFIX or pdFIX by the one‐stage clotting assay. However, minimizing interlaboratory variability is vital for optimizing future patient care.

## 1 INTRODUCTION

In clinical coagulation laboratories, the one‐stage clotting assay is the preferred method for measuring factor IX (FIX) activity in plasma,^1^ as well as the activity of FIX products that are used for the treatment of hemophilia. This method is based on the activated partial thromboplastin time (aPTT), for which a variety of activators, phospholipid components, calibrator plasmas, and instruments are commercially available. Although the calibrator plasmas are traceable to an international standard for FIX activity (commissioned by the World Health Organization), the diversity of available reagents and instruments has resulted in significant assay variability, particularly reported for recombinant FIX (rFIX) products.^2^, ^3^, ^4^, ^5^, ^6^ Furthermore, there is greater variability for some of the newer extended half‐life FIX products^7^ compared with the conventional rFIX products. For example, a PEGylated FIX product is incompatible with all commonly used silica‐based aPTT reagents for the one‐stage clotting assay, resulting in pronounced overestimation of plasma FIX levels.^8^ Such assay variability is of particular concern when accurate clinical monitoring is required (eg, during surgical procedures). Therefore, manufacturers of all FIX products must justify their choice of assay used for potency assignment and, when introducing a new FIX product into clinical practice, ensure that it can be measured with acceptable accuracy and reliability using one‐stage clotting assays, employing all commonly used aPTT reagents across multiple laboratories.^3^

ALPROLIX^®^ (Bioverativ Therapeutics Inc, an affiliate of Sanofi) is a long‐acting, recombinant FIX Fc fusion protein (rFIXFc) approved for the treatment of hemophilia B.

The potency of rFIXFc was assigned using the Siemens Actin aPTT reagent in a one‐stage clotting assay against a rFIXFc reference standard that was calibrated to the World Health Organization FIX concentrate standard by the same method.^9^ Previously, variability of the one‐stage clotting assay using different aPTT reagents was assessed for rFIXFc in a field study at 30 clinical hemostasis laboratories in seven countries, not including Japan.^9^ In that study, the FIX activities of three different concentrations of rFIXFc or rFIX (BENEFIX^®^; Wyeth Pharmaceuticals Inc) spiked into human hemophilic donor plasma with no detectable FIX activity (<0.5%) were measured using the laboratories’ routine one‐stage clotting assay in a blinded manner. The field study showed that aPTT reagents, instruments, and reference standards varied between laboratories and revealed interlaboratory variability for both rFIXFc and rFIX, especially at lower concentrations. However, significant inaccuracy of rFIXFc compared with rFIX was only observed with the Stago C. K. Prest^®^ kaolin reagent, which underestimated rFIXFc activity by up to 50%. The field study concluded that, based on spike recoveries (percentage of nominal activity based on label potency), and compared with the accuracy achieved when measuring the comparator rFIX product, most participating laboratories measured rFIXFc activity with acceptable accuracy and reliability using routine one‐stage assay methods.^9^

No reported study has measured rFIXFc activity under blinded conditions at Japanese clinical laboratories that routinely measure FIX activities by one‐stage clotting assays. In Japan, there are currently approximately 20 aPTT reagents available, the most widely used being Data‐fi APTT, Thrombo‐check APTT, Thrombo‐check APTT‐SLA, Coagpia APTT‐N, Actin‐FSL, and HemosIL SynthASil. Kaolin‐based reagents are rarely, if at all, used for one‐stage clotting assays in Japan.

Because the aPTT reagents, instruments, and reference standards used in Japan may differ from those in the field study discussed above, we conducted a separate field study in Japan. The “J‐Field Study” aimed to evaluate the accuracy of measuring rFIXFc activity with the one‐stage clotting assays used at major Japanese hemophilia treatment centers and commercial laboratories.

## 2 MATERIALS AND METHODS

### 2.1 Preparation of the J‐Field Study kits

Spiked samples of rFIXFc, rFIX, and pdFIX were prepared and distributed as outlined in Appendix S1.

### 2.2 Study design

This study was a multicenter, assessor‐blinded, prospective study performed in 18 clinical laboratories throughout Japan that routinely measure FIX activity with a one‐stage clotting assay. The previous rFIXFc field study showed that the intralaboratory coefficient of variation (CV) averaged 4%‐8% for both rFIX and rFIXFc across all concentrations.^9^ Since intralaboratory variability was a minor contributor to the overall (interlaboratory) variability in FIX measurements, we asked the laboratories to test each sample only once for this study, thereby rendering the study design more representative of actual clinical sample testing.

### 2.3 Data analysis

Data from each laboratory and the accreditation status of each laboratory were listed and summarized descriptively. Pearson correlation coefficients were calculated to assess linearity and interlaboratory variability of FIX activity was calculated as CV (%) for each product and concentration. Spike recovery (percent of nominal activity based on actual labeled vial potency) was calculated by the measured FIX activity divided by the nominal activity × 100. The measured rFIXFc activity was compared graphically with those of rFIX and pdFIX and for the types of aPTT reagents (ellagic acid, polyphenol, and silica). The statistical analyses were conducted using SAS^®^ 9.3 (SAS Institute, Inc).

## 3 RESULTS

### 3.1 Study laboratories

This study was conducted from November to December 2015 and 18 laboratories participated, including medical institutions and commercial laboratories. One site performed two different one‐stage clotting assay procedures (shown as Labs 03 and 19 in figures), yielding 19 laboratory procedures in the analyses.

### 3.2 Measured FIX activities of rFIXFc compared with pdFIX and rFIX

**Figure 1:**
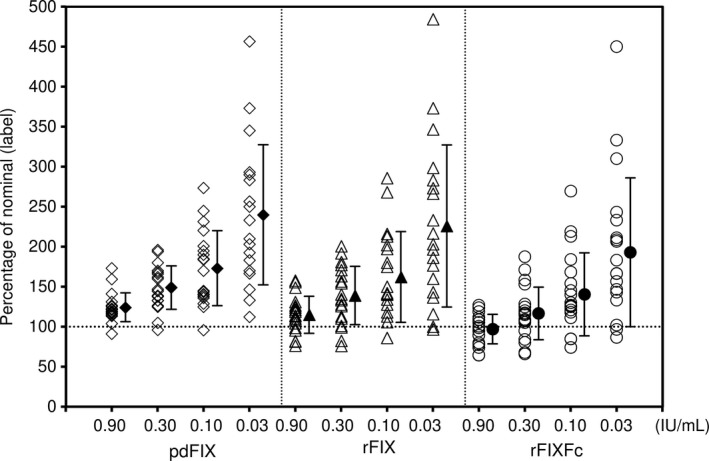
Percentage of factor IX (FIX) activity vs nominal label activity categorized by the FIX product. Open marks indicate the FIX activity for 19 laboratories. Closed marks and bars indicate the mean and standard deviation, respectively. pdFIX, plasma‐derived factor IX; rFIX, recombinant factor IX; rFIXFc, recombinant factor IX Fc fusion protein. Percentage of nominal (label) was calculated by the measured FIX activity divided by the label potency of the sample × 100

The FIX activities and CV (%) of each product are summarized in Table 1. The spike recoveries are shown in Figure 1. The interlaboratory CV for the one‐stage clotting assay was similar between rFIXFc and rFIX at all concentrations, ranging from 19% at 0.90 IU/mL to 48% at 0.03 IU/mL for rFIXFc and from 20% at 0.90 IU/mL to 45% at 0.03 IU/mL, respectively, for rFIX. The results provided by the three commercial laboratories alone were representative of the FIX activity measurements and variability seen among all laboratories (data not shown). The CV of pdFIX was slightly lower at all FIX concentrations than for the two rFIX products, ranging from 15% to 37%. Pearson correlation coefficients of the mean FIX activity from the 19 laboratories in the range of 0.03 IU/mL to 0.90 IU/mL were 0.9537, 0.9522, and 0.9741 for rFIXFc, rFIX, and pdFIX, respectively. A likely cause for the differences in the variability of FIX activity observed among these products might be different reactivities of each product in the various aPTT reagents.^4^ The average spike recovery of rFIXFc samples was closer to the labeled activity at each concentration level compared with sample measurements for rFIX and pdFIX, which were overestimated to a greater degree at each level (Table 1). The majority of laboratories increasingly overestimated the FIX label activity at decreasing FIX levels; this resulted in, on average, a twofold overestimation of FIX activity at the nominally 0.03 IU/mL level irrespective of the FIX product (Figure 1). Although the label activities of the three products have been measured by approved methods, a direct comparison of the nominal FIX activity between the products was not possible because our laboratory has not verified the activities for the three products.

**Table 1:** Summary statistics of FIX activities by the FIX products (n = 19)

FIX product	Label activity (IU/mL)	Mean FIX activity (IU/mL)	CV (%)
rFIXFc	0.03	0.0579	48.2
	0.10	0.1405	36.9
	0.30	0.3497	28.2
	0.90	0.8721	19.1
rFIX	0.03	0.0678	44.9
	0.10	0.1621	35.0
	0.30	0.4169	26.3
	0.90	1.0333	20.2
pdFIX	0.03	0.0720	36.5
	0.10	0.1731	27.0
	0.30	0.4465	18.2
	0.90	1.1178	14.6

Note

The CV (%) is calculated by dividing standard deviation by mean FIX activity multiplied by 100.

Abbreviations: CV, coefficient of variation; FIX, factor IX; pdFIX, plasma‐derived factor IX; rFIX, recombinant factor IX; rFIXFc, recombinant factor IX Fc fusion protein.

**Figure 2:**
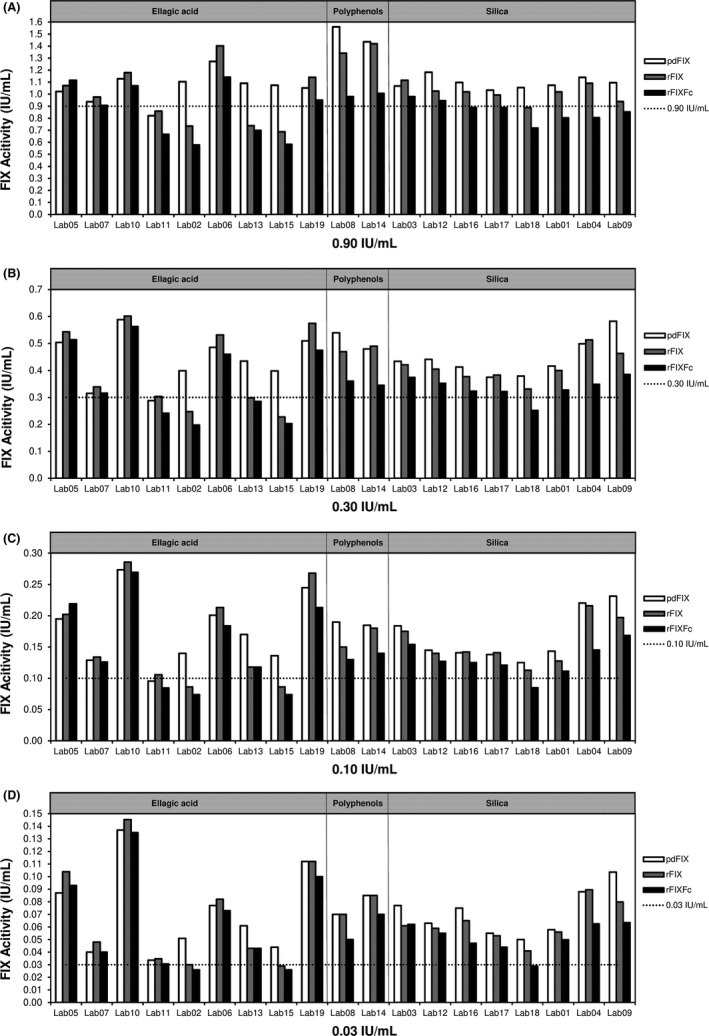
Percentage of FIX activity to nominal in individual laboratories at concentrations of 0.90 IU/mL (A), 0.30 IU/mL (B), 0.10 IU/mL (C), and 0.03 IU/mL (D). pdFIX, plasma‐derived factor FIX; rFIX, recombinant factor IX; rFIXFc, recombinant factor IX Fc fusion protein. The dotted line shows 100% nominal at each concentration

The mean (standard deviation) FIX activities by type of activator in the aPTT reagent are shown for each FIX product in Table 2 and Figure 2. Irrespective of aPTT reagent and instrument type, mean activities of rFIX and pdFIX were mostly higher than the nominal concentrations. On the other hand, mean activities of rFIXFc were closer to the nominal values, especially at the 0.90 IU/mL level.

**Table 2:** Summary of FIX activities and CV (%) of the FIX products by activating reagents

FIX product	aPTT reagent activator	Label activity (IU/mL)	Mean (SD) activity (IU/mL)		CV (%)
rFIXFc	Ellagic acid (n = 9)	0.03	0.0630	(0.0392)	62.2
		0.10	0.1513	(0.0721)	47.6
		0.30	0.3617	(0.1417)	39.2
		0.90	0.8563	(0.2289)	26.7
	Polyphenols (n = 2)	0.03	0.0600	(0.0141)	23.6
		0.10	0.1350	(0.0071)	5.2
		0.30	0.3525	(0.0106)	3.0
		0.90	0.9925	(0.0177)	1.8
	Silica (n = 8)	0.03	0.0516	(0.0118)	22.8
		0.10	0.1296	(0.0261)	20.2
		0.30	0.3356	(0.0410)	12.2
		0.90	0.8599	(0.0842)	9.8
rFIX	Ellagic acid (n = 9)	0.03	0.0698	(0.0425)	61.0
		0.10	0.1665	(0.0774)	46.5
		0.30	0.4075	(0.1521)	37.3
		0.90	0.9760	(0.2424)	24.8
	Polyphenols (n = 2)	0.03	0.0775	(0.0106)	13.7
		0.10	0.1650	(0.0212)	12.9
		0.30	0.4800	(0.0141)	2.9
		0.90	1.3800	(0.0566)	4.1
	Silica (n = 8)	0.03	0.0631	(0.0154)	24.4
		0.10	0.1565	(0.0358)	22.9
		0.30	0.4117	(0.0558)	13.5
		0.90	1.0112	(0.0740)	7.3
pdFIX	Ellagic acid (n = 9)	0.03	0.0714	(0.0352)	49.3
		0.10	0.1761	(0.0579)	32.9
		0.30	0.4359	(0.0969)	22.2
		0.90	1.0552	(0.1256)	11.9
	Polyphenols (n = 2)	0.03	0.0775	(0.0106)	13.7
		0.10	0.1875	(0.0035)	1.9
		0.30	0.5100	(0.0424)	8.3
		0.90	1.4975	(0.0884)	5.9
	Silica (n = 8)	0.03	0.0712	(0.0183)	25.7
		0.10	0.1660	(0.0407)	24.5
		0.30	0.4425	(0.0686)	15.5
		0.90	1.0932	(0.0481)	4.4

Note

The CV (%) is calculated by dividing SD by mean FIX activity.

Abbreviations: aPTT, activated partial thromboplastin time; CV, coefficient of variation; FIX, factor IX; pdFIX, plasma‐derived factor IX; rFIX, recombinant factor IX; rFIXFc, recombinant factor IX Fc fusion protein; SD, standard deviation.

**Figure 3:**
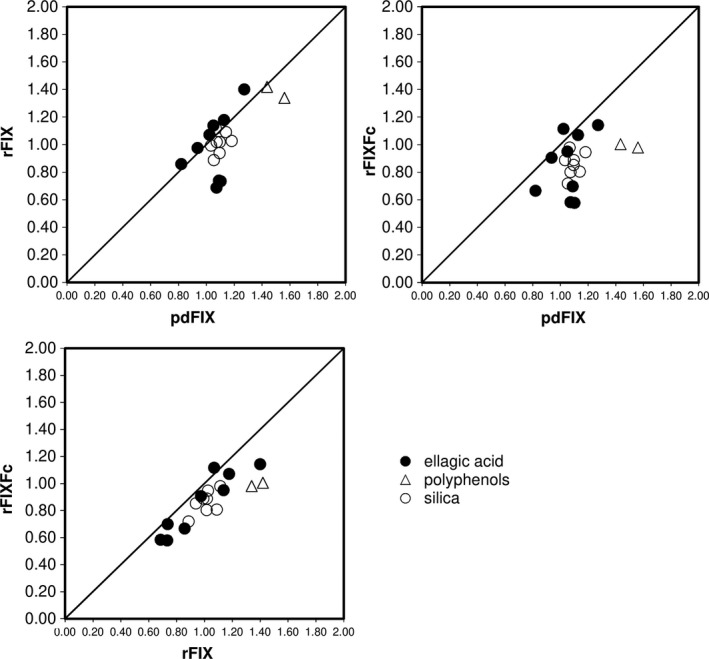
Scatter plot of factor IX activities between each product at 0.9 IU/mL. pdFIX, plasma‐derived factor IX; rFIX, recombinant factor IX; rFIXFc, recombinant factor IX Fc fusion protein. The diagonal line shows the positive correlation line

The silica‐based reagents used by participants in this study showed near nominal recovery at the 0.90 IU/mL level, and more consistent agreement was observed between laboratories using silica‐based reagents than in laboratories using ellagic acid‐based reagents (Figure 3). The results with silica‐based reagents were generally in close agreement and near a 1:1 ratio between the three drug products. On the other hand, the distribution of results from ellagic acid‐based reagents was more widely spread due to larger interlaboratory variability. The correlations between the two recombinant products were stronger than each recombinant product vs pdFIX. In three of the nine laboratories using ellagic acid‐based reagents, the activity of pdFIX was approximately 30% higher than that of rFIXFc or rFIX, while the other laboratories did not observe consistent differences between pdFIX and two rFIX activities (Figure 2). However, due to the large variability between laboratories using the same ellagic acid reagents, it is difficult to compare the accuracy of individual reagents. Nor can we determine with certainty the relative spike recovery of any product using ellagic acid vs silica‐based reagents.

## 4 DISCUSSION

The J‐Field Study evaluated the accuracy of measuring rFIXFc activity with the one‐stage clotting assays using rFIX and pdFIX as comparators. This was the first study in Japan that measured rFIXFc activity under blinded conditions using the same sample kits at multiple laboratories, including medical institutions and commercial laboratories, that routinely measure FIX activity throughout Japan. A previous field study that included primarily US and European laboratories showed higher reagent‐dependent variability for rFIXFc compared with rFIX.^9^ However, in the current study, the interlaboratory variability of rFIXFc was not significantly different from that of rFIX. Moreover, the mean spike recovery for rFIXFc was generally comparable for aPTT reagents using ellagic acid, polyphenol, or silica as activators; while in the previous study, the reported activity was generally lower and more variable with silica‐based reagents. The differences between the two studies may be primarily due to regional differences in availability and use of aPTT reagents. For example, kaolin‐based reagents were not used in our field study as they are rarely used for one‐stage assays in Japan, but were associated with lower recoveries in the earlier field study.^9^ Some brands of ellagic acid‐based reagents used in our study were the same as those reported in the previous study.^9^ However, one major brand of ellagic acid‐based reagent used by five laboratories in our study is unique to the Japanese market. In the case of silica‐based reagents, eight laboratories in our study used three brands, while the previous study included six brands in 17 laboratories. Importantly, the silica‐based reagents that resulted in significant underestimation of rFIXFc compared with rFIX in the previous research are rarely used in Japan and were not included in the current study. Thus, the consistent performance and lower interlaboratory variability of silica‐based reagents seen in our study are likely the result of preferential use of reagents in Japan that coincidentally provide more accurate results for rFIXFc than what were observed previously in the international study.

In our study, the laboratories using ellagic acid‐based reagents demonstrated noticeably higher interlaboratory CVs, even when using the same brand of reagents. In particular, some laboratories reported significantly lower activities for rFIX and rFIXFc than for pdFIX, while others did not. The potential impact of other variables on these assay results, including instrument differences, source of calibrator plasma, number of calibrator dilutions, and number of dilutions tested per sample, or the use of multiple calibration curves over the assay range were considered; however, no methodological differences that could account for discrepant laboratory results were found.

The mean FIX activity of rFIXFc as determined by the one‐stage clotting assay was overestimated except at the 0.90 IU/mL spike level. A pronounced lack of dilution linearity was observed for all three FIX products and has been reported previously for rFIXFc and rFIX.^9^ Since this lack of dilution linearity was also observed for pdFIX in the current study suggests that this may not be an inherent property of rFIX products, but more likely a discrepancy between the dilution of the assay calibrator, typically performed in buffer or saline on to the instrument, and the samples provided in FIX‐deficient plasma. In addition, the four concentrations of the sample kits in this study were diluted serially, which may amplify any potential dilution error as the concentration decreases. Nonlinearity of the FIX assay may be a concern when physicians attempt to optimize dosing regimens of extended half‐life FIX products for each patient based on a pharmacokinetic assessment, as inaccurate and overestimated FIX activity at low levels may impact the prediction of some pharmacokinetic parameters such as terminal half‐life and trough levels.

A limitation of this study is the use of human hemophilic donor plasma spiked with FIX products to evaluate FIX activity levels. It is possible that results from postinfusion samples taken from people with hemophilia receiving various FIX products would be different as data on the in vivo integrity of modified coagulation factors have not been reported. Another potential limitation to be noted in this study is the relatively low number of enrolled laboratories. The exact number of laboratories that routinely measure FIX activity in Japan is not known. However, the laboratories that participated in our study were the major Japanese hemophilia treatment centers and commercial laboratories that routinely measure FIX activities of samples from patients with hemophilia. Generally, Japanese commercial laboratories play an important role in the hemophilia field, as they test for the clinics or hospitals throughout Japan that do not have a laboratory or the facilities required to measure FIX activities. By enrolling both the medical institutions and commercial laboratories, we assumed that the majority of laboratories measuring FIX activities throughout Japan would be included and the results could be considered representative of Japanese procedures and common practice. Our data show that the commercial laboratories that participated in this study provided similarly reliable FIX results as hospital‐associated laboratories. Although we stratified our analysis for types of aPTT reagents, we did not strictly assess the differences between types of aPTT reagents because the aim of this study was not to compare the differences between activators, nor individual brands of aPTT reagents, but to assess the ability of existing FIX one‐stage clotting assays to measure rFIXFc activity. More detailed studies would be needed to assess the impact of differences between types of aPTT reagents used in Japanese clinical practice for measuring FIX activities by the one‐stage clotting assay. Lastly, our data did not reveal a correlation between assay performance and the type of laboratory, participation in proficiency studies, or the accreditation status.

The J‐Field Study evaluated the accuracy of measuring rFIXFc activity with the one‐stage clotting assays at major Japanese hemophilia treatment centers and commercial laboratories using their routine assay procedures. Compared with rFIX and pdFIX, interlaboratory variability of rFIXFc activity was not significantly different when using the existing assay methods. Results from this field study show that measurement of rFIXFc activity is similar to that of rFIX or pdFIX by the one‐stage clotting assay in most participating laboratories. It should, however, be noted that the values of some laboratories differed considerably from the consensus results. Further harmonization of the types of reference standards, aPTT reagents, as well as analytical methods and quality control procedures, would likely improve the accuracy and reduce interlaboratory variability of the one‐stage clotting assay,^10^ which is vital for optimizing future patient care.

## CONFLICT OF INTEREST

Katsuyuki Fukutake has received grants from Baxalta, Bayer, Pfizer, CSL Behring, Novo Nordisk, Biogen, Kaketsuken, Japan Blood Products Organization, and Ortho Clinical Diagnostics; personal fees from Baxalta, Bayer, Pfizer, CSL Behring, Novo Nordisk, Biogen, Kaketsuken, SRL Inc, LSI Medience, Roche Diagnostics, Siemens, Sekisui Medical, Fujirebio Inc, Abbott, Torii Pharmaceuticals, Octapharma, Chugai Pharmaceutical Co. LTD, and BioMarin; and other fees from CIMIC outside the submitted work. Tomomi Kobayashi is a current employee of Bioverativ Japan Ltd. Jurg M. Sommer is a former employee of and current consultant for Bioverativ, a Sanofi company. Toshiyuki Hirakata is a former employee of Biogen Japan Ltd.
