# Supplementary material for: Recombinant FIX Fc fusion protein activity assessment with the one‐stage clotting assay: A multicenter, assessor‐blinded, prospective study in Japan (J‐Field Study)
Source: Int J Lab Hematol. 2019 Dec 10;42(2):162–9. doi: 10.1111/ijlh.13133 (PMC7078902; doi:10.1111/ijlh.13133)
Supplement: Supplementary file 1 [file IJLH-42-162-s001.docx]

**Appendices**

**Appendix A**

To prepare the field study samples, ALPROLIX^®^, BENEFIX^®^, and MONONINE^®^ (pdFIX; CSL Behring LLC, Kankakee, IL, USA) were spiked into congenital FIX-deficient plasma from a single donor (George King Bio-Medical, Overland Park, KS, USA) with no measurable FIX activity (<0.005 IU/mL) to reach 0.90 IU/mL based on the actual labeled vial potency. The 0.90 IU/mL FIX plasma was serially diluted to prepare concentrations of 0.30, 0.10, and 0.03 IU/mL, yielding 12 samples per the J-Field Study kit. The kits were stored at <–70°C until distribution to participating laboratories. The kits were sent to the laboratories with blinded FIX product names and concentrations. FIX activity of the 12 provided samples using a FIX one-stage clotting assay was measured in accordance with the laboratory’s routine procedure. Previous rFIXFc assay validation studies had shown excellent frozen sample stability and no significant loss of rFIXFc activity through 2 freeze-thaw cycles when diluted into human plasma (unpublished).

The following information was collected from the laboratories: FIX activity (IU/mL) of the 12 sample measurements, the specific coagulation analyzer, aPTT reagents, activator in the reagents, standard plasma, number of standard curve points, calibration frequency, and FIX-deficient plasma used for the assay. An independent company received the data collection forms for data management and analysis.

**Appendix B** Instruments and activated partial thromboplastin time (aPTT) reagents used in the laboratories in this study.

| Laboratory ID | aPTT reagent | Instrument | Calibrator  (standard plasma) | Activating reagent |  |
| --- | --- | --- | --- | --- | --- |
| Lab 02 | Thrombo-check  APTT-SLA | CS-2000i | SIEMENS | ellagic acid |  |
| Lab 05 | Actin-FSL | CS-2400 | SIEMENS | ellagic acid |  |
| Lab 06 | Thrombo-check  APTT-SLA | CS-5100 | SIEMENS | ellagic acid |  |
| Lab 07 | Data-fi APTT(ACTIN) | CS-2100i | SIEMENS | ellagic acid |  |
| Lab 10 | Data-fi APTT(ACTIN) | CP-3000 | SIEMENS | ellagic acid |  |
| Lab 11 | Data-fi APTT(ACTINFS) | Coapresta 2000 | Sysmex | ellagic acid |  |
| Lab 13 | Thrombo-check  APTT-SLA | Coagrex-800 | Sysmex | ellagic acid |  |
| Lab 15 | Thrombo-check  APTT-SLA | CS-5100 | SIEMENS | ellagic acid |  |
| Lab 19 | Thrombo-check  APTT-SLA | CS-5100 | In-house pooled plasma | ellagic acid |  |
| Lab 08 | STA Cephascreen (APTT) | STA-R Evolution | DIAGNOSTICA STAGO | polyphenols |  |
| Lab 14 | STA Cephascreen (APTT) | STA-R Evolution | In-house pooled plasma | polyphenols |  |
| Lab 01 | Platelin-LS II | COAGTRON-350 | GEORGE KING  Bio-Medical, Inc. | silica |  |
| Lab 03 | HemosIL SynthASiL | ACL TOP500 | Instrumentation Laboratory | silica |  |
| Lab 04 | Platelin-LS II | Coagrex-800 | SIEMENS | silica |  |
| Lab 09 | Platelin-LS II | CS-2100i | SIEMENS | silica |  |
| Lab 12 | HemosIL SynthASiL | ACL TOP700 | Instrumentation Laboratory | silica |  |
| Lab 16 | HemosIL SynthASiL | ACL TOP | In-house pooled plasma | silica |  |
| Lab 17 | HemosIL SynthASiL | ACL TOP700 | Instrumentation Laboratory | silica |  |
| Lab 18 | Pathromtin SL | CS-5100 | Sysmex | silica |  |

[Table footnote]: A total of eight aPTT reagents with three kinds of activators were used: ellagic acid (four brands in nine laboratories), polyphenols (one brand in two laboratories), and silica (three brands in eight laboratories). Kaolin-based reagents were not used by any laboratory. A total of 12 different instruments were used by the 19 laboratories (Appendix A). The number of standard curve points ranged from four to 10 points in all laboratories except one that used two standard curves with six concentrations, using three points in the low-assay range and three points in the high-assay range. The standard curve samples were prepared fresh on day of analysis. The instruments were most frequently calibrated when the lot number of the aPTT reagents and/or FIX-deficient plasma was changed (nine out of 19 laboratories, 47.4%). Seven of 19 laboratories (36.8%) calibrated their instruments before each assay run. Among the 19 laboratories, five (including the three commercial laboratories) provided a certificate of accreditation in the year of 2015: four laboratories are certified based on ISO 15189 by the Japan Accreditation Board, and one laboratory is accredited by the College of American Pathologists.
